# Othello syndrome as an early manifestation of Alzheimer's disease: an anatomoclinical case study

**DOI:** 10.1590/1980-5764-DN-2025-0461

**Published:** 2026-08-21

**Authors:** Paulo Roberto de Brito Marques, Maria Luiza Vasconcelos Montenegro, Marcela Maria Cabral Ribeiro

**Affiliations:** 1 Universidade de Pernambuco, Faculdade de Ciências Médicas, Recife PE, Brazil.; 2 Universidade de Pernambuco, Hospital Universitário Oswaldo Cruz, Recife PE, Brazil.

**Keywords:** Alzheimer's Disease, Dementia, Delusions, Geriatric Psychiatry, Jealousy, Neuropsychiatry, Doença de Alzheimer, Demência, Delusões, Psiquiatria Geriátrica, Ciúme, Neuropsiquiatria

## Abstract

Othello syndrome (OS) is characterized by pathological jealousy toward one's spouse, usually marked by delusions of infidelity. We report the first case of OS in Brazil, similar to the case described by Alzheimer in 1906, in a 54-year-old right-handed patient diagnosed with Alzheimer's disease (AD). The diagnostic workup included a clinical history and a positive family history of dementia, the Clinical Dementia Rating (CDR) scale, the Edinburgh Handedness Inventory, and the Mini-Mental State Examination (MMSE). Electroencephalography revealed slow waves predominantly in the bilateral temporo-frontal regions. Cranial computed tomography showed diffuse cortico-subcortical atrophy compatible with the patient's age, as well as bilateral globus pallidus calcifications. Cerebral single-photon emission computed tomography revealed hypoperfusion in the medial frontal and bilateral temporal regions, more pronounced on the left — particularly in the inferomedial portion of the left temporal lobe — along with mild supratentorial ventricular dilatation. Postmortem examination revealed moderate bilateral fronto-temporo-parietal atrophy, and microscopy showed the presence of senile plaques and neurofibrillary tangles. We report an unusual case of OS as the initial clinical manifestation, later diagnosed as AD.

## RESUMO

A síndrome de Otelo (SO) caracteriza-se por um ciúme patológico em relação ao cônjuge, geralmente marcado por delírios de infidelidade. Relatamos o primeiro caso de SO no Brasil, semelhante ao caso descrito por Alzheimer em 1906, em um paciente destro de 54 anos diagnosticado com doença de Alzheimer. A investigação diagnóstica incluiu a anamnese clínica e o histórico familiar positivo para demência, a Escala de Avaliação Clínica da Demência (CDR), o Inventário de Lateralidade de Edimburgo e o Miniexame do Estado Mental (MEEM). O eletroencefalograma revelou ondas lentas difusas predominantemente nas regiões temporofrontais bilaterais. A tomografia computadorizada do crânio mostrou atrofia córtico-subcortical difusa compatível com a idade do paciente, bem como calcificações bilaterais do globo pálido. A tomografia computadorizada por emissão de fóton único (SPECT) cerebral revelou hipoperfusão nas regiões frontal medial e temporal bilateral, mais pronunciada à esquerda — particularmente na porção ínfero-medial do lobo temporal esquerdo — junto com leve dilatação ventricular supratentorial. O exame *post-mortem* revelou atrofia fronto-têmporo-parietal bilateral moderada, e a microscopia mostrou a presença de placas senis e emaranhados neurofibrilares. Relatamos um caso raro de SO como manifestação clínica inicial, posteriormente diagnosticada como DA.

## INTRODUCTION

Othello Syndrome (OS) is characterized by an unusual clinical presentation involving irrational and morbid jealousy, in which the patient experiences delusional thoughts of infidelity directed toward their partner, despite the absence of any real evidence for such suspicions^1^. In 1906, Alois Alzheimer described the case of Auguste Deter, who exhibited jealousy towards her husband, along with a rapid decline in recent memory and marked psychosocial impairment, demonstrating that delusional jealousy is a frequent problem in patients with dementia^2^. The term "delusional jealousy" became known as *Othello Syndrome,* which was coined by Todd and Dewhurst in 1955^1^. The behavior of accusing someone of infidelity develops in parallel with cognitive deterioration: in the early stages of dementia, the symptoms are more pronounced at night; as time passes, the accusations become more frequent during the day^3^. It is believed that dysfunction of the right hemisphere, especially the parietal lobe, may be associated with delusional jealousy in patients with early-onset AD^4^.

OS occurs more frequently in men with an average age of 58 years, with an average duration of 40 months. Etiologies included primary psychiatric disorders (22%), other medical conditions (52%), and medications or other substances (26%). Delusional disorder, cerebrovascular accident, and dopaminergic agonists were the most common etiologies, respectively, in these groups^5^. OS is often secondary to other clinical conditions, such as with or without the use of dopamine agonists in Parkinson's disease^6-8^, after a stroke in the right cerebral hemisphere^9^, brain infection secondary to a tuberculoma^10^, normal pressure hydrocephalus^11^, chronic alcoholism^12^, and AD (15%)^13-15^.

This study describes a rare case of OS that occurred as an early clinical presentation of AD.

## METHODS

A clinical case of OS in a right-handed patient with AD was diagnosed. Clinical and family history, the Edinburgh Handedness Inventory^16^, Clinical Dementia Rate (CDR)^17^, Measurement of Functional Activities in Older Adults in the Community (Pfeffer)^18^, the Mini-Mental State Examination (MMSE)^19^, Computer Tomography (CT) of the brain, Single-photon emission computed tomography (SPECT) scan, and postmortem study were carried out according to the isocortical stage, or Braak stage V–VI, the criteria of the Consortium to Establish a Registry for Alzheimer's Disease (CERAD)^20^. Immunohistochemical reactions for anti Tau (Pt), anti-β amyloid (βA), and anti-ubiquitin (U) proteins were used to evaluate the neurofibrillary tangles and senile plaques, and each exceeding 20 per 100X. The authors declare that they have adhered to their institution's protocols for the publication of patient data. This study was approved by the Research Ethics Committee of Oswaldo Cruz University Hospital (CAAE: 39103420.1.0000.5192). Written consent for publication was obtained from the patient's family.

## CASE REPORT

A 74-year-old married White male with a degree in accounting and retired was admitted to the Cognitive and Behavioral Neurology Clinic at the University of Pernambuco in 1996. Approximately twenty years earlier, the patient had begun to exhibit persistent obsessive jealousy. According to his wife, until the age of 54, he had never expressed or shown any signs of jealousy. The symptoms began when he started questioning her about the delays in returning home from the supermarket. Over time, his jealousy progressively intensified, leading him to forbid men, including family members and cleaning staff, from entering the house. His wife reported that during their arguments, while she cried and felt humiliated, her husband remained emotionally indifferent, as if discussing an ordinary matter. After 15 years of intermittent jealousy-fueled accusations that had progressively escalated, the case eventually reached the courts as a request for separation. In the absence of any prior complaints against the husband, the judge recommended postponing the ruling, interpreting the behavior as a potentially temporary phase in the marriage. Two years after the judge denied the separation, the patient continued to show jealousy toward his wife, this time directed at his eldest son, whom he claimed was not his biological son. When presented with the son's identity card, the patient stated that it was easy to place anyone's name on such a document. Later, the patient assaulted his grandson for hugging his grandmother. At that point, both his wife and the household staff realized that the husband was no longer mentally healthy. The episodes of jealousy began sporadically and indirectly, gradually increasing over the years. The jealousy manifested as arguments between the couple, and the children became aware of the issue at the time of the court proceedings. The patient was not taken to any other physician because his wife believed it was merely jealousy, although she found it very strange; however, this was how she perceived the situation. Once the possibility of a mental illness was recognized, attention was redirected toward the husband as the individual affected. Following the onset of delusional symptoms, the patient's cognition and functional abilities declined markedly, and he became dependent on assistance for routine household activities.

**Figure 1:**
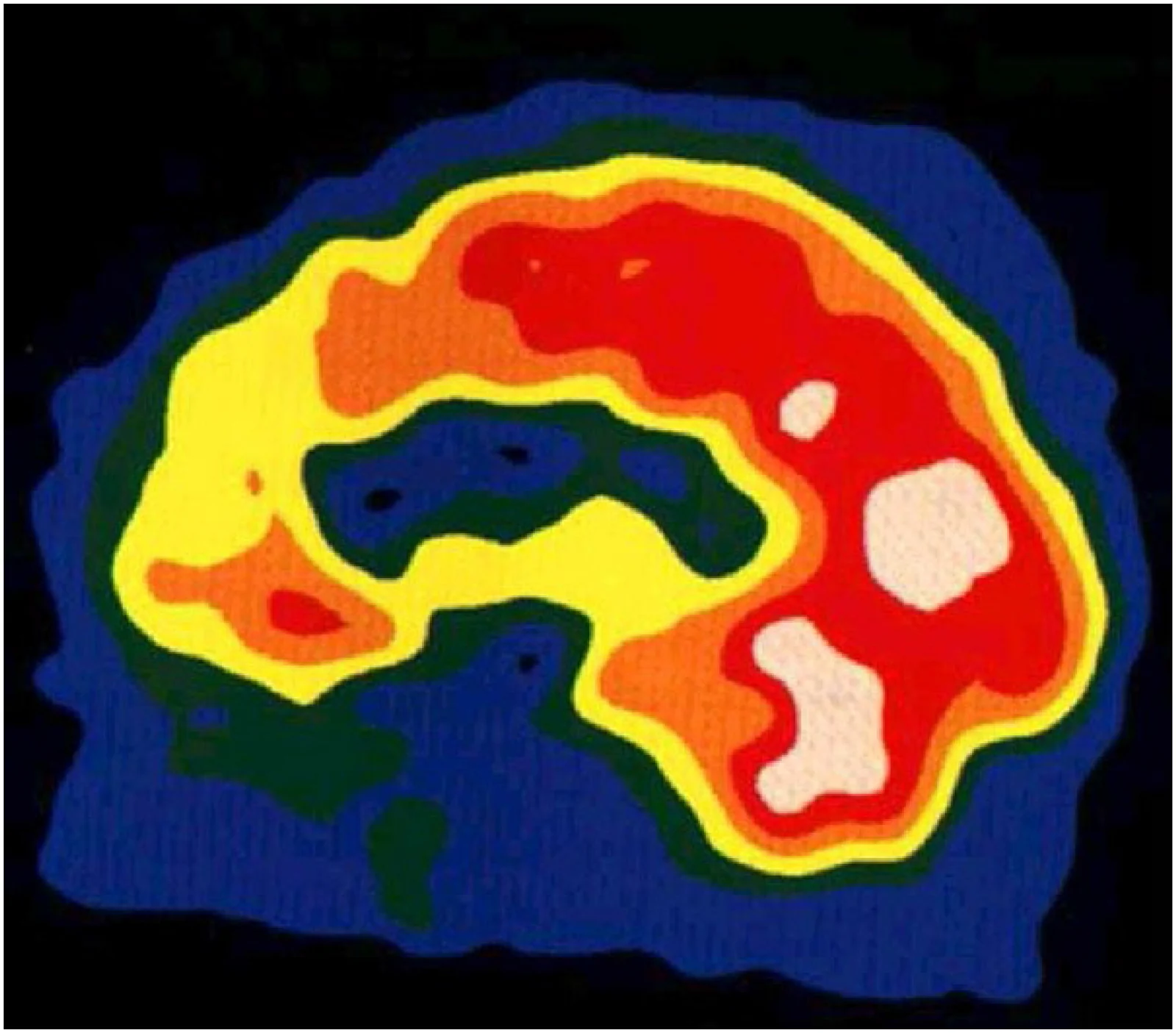
Cerebral SPECT reveals moderate to severe hypoperfusion in the frontal lobe.

The patient was not taking any medication that could induce this delirium. The patient had mild diabetes mellitus. The patient's mother, maternal grandmother, four female cousins, and one male cousin died of dementia. During the first consultation, it was observed that the patient was right-handed and showed signs of moderately severe to severe dementia. The CDR score was 2/3, the Measurement of functional activities in older adults in the community (Pfeffer et al. 1982) score was (22/30), and his MMSE score was (11/30). Laboratory tests for dementia screening were normal. The electroencephalogram showed disorganized background activity and diffuse rhythmic theta/delta slowing, predominantly in the bilateral frontotemporal regions. CT of the brain revealed diffuse cortico-subcortical atrophy, most evident in the frontotemporal lobes, as well as calcifications in the bilateral globus pallidus and moderate widening of the Sylvian fissures bilaterally. SPECT scan of the brain revealed hypoperfusion in the medial frontal and bilateral temporal regions, more intense on the left, mainly in the inferomesial portion of the left temporal lobe, as well as mild dilation of the supratentorial ventricular system (Figure 1).

### Follow-up

**Figure 2:**
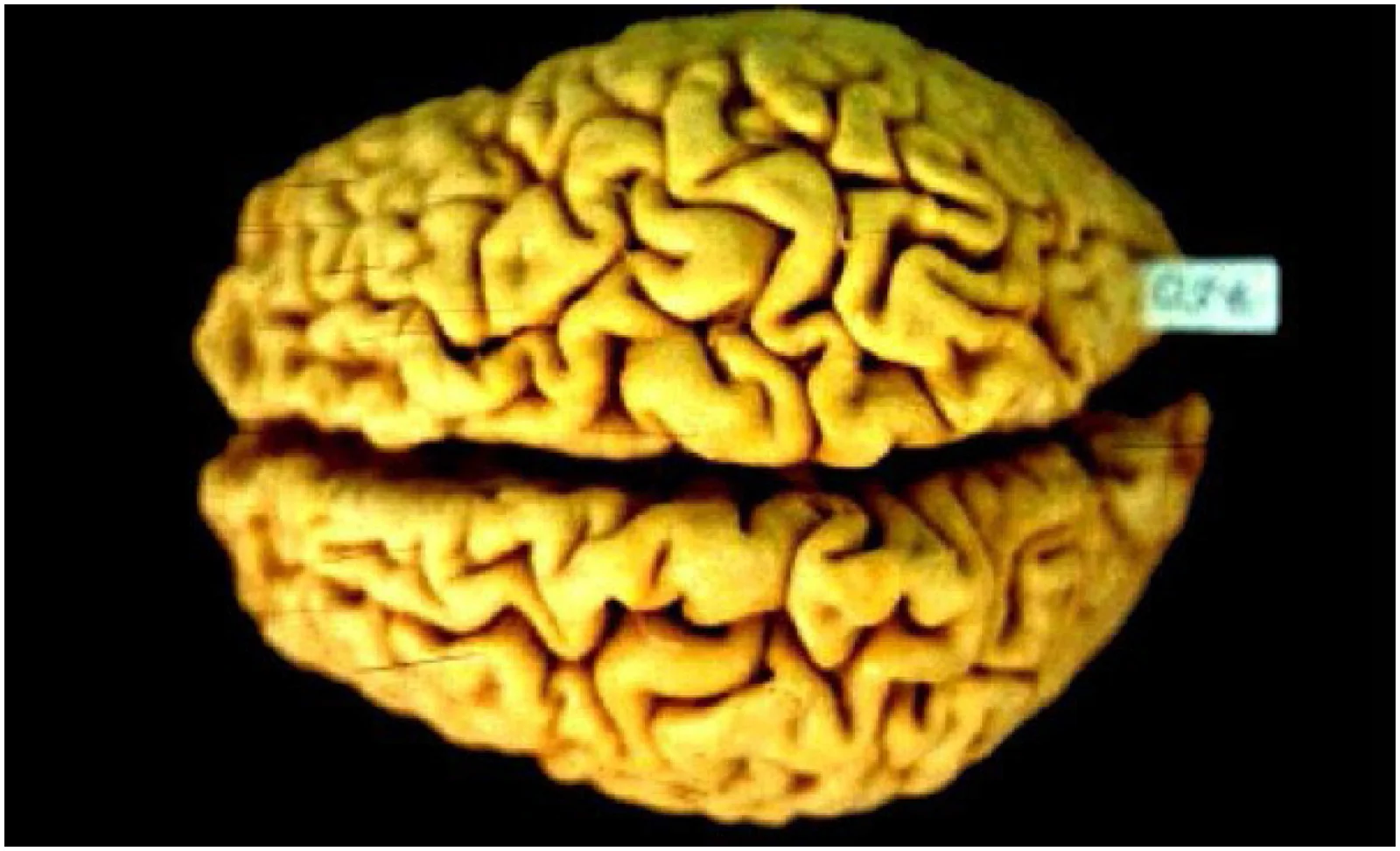
Macroscopic appearance of the brain with moderate bilateral frontal lobe atrophy.

The patient was using haloperidol, piracetam, amitriptyline, and dihydroergocristine. In November 1997, the patient scored 0/30 on the MMSE and was unable to engage in comprehensible dialogue. Although some spontaneous words were partially preserved, his speech, when questioned, was predominantly characterized by neologisms. In February 1998, the patient began keeping his eyes closed, and his speech became unintelligible most of the time, although he was still able to ambulate freely with support. He presented with immobility syndrome, being bedridden for the last two years of his life, undergoing motor and respiratory physiotherapy three times a week. In 2000, the patient was found lifeless in the morning and underwent an autopsy. His brain weighed 1,200 g and, macroscopically, showed convolutions with altered relief due to diffuse gyral atrophy with bilateral fronto-parieto-temporal accentuation. No abnormalities were observed in the major basal vessels, but coronal sections revealed bilateral hippocampal parenchymal atrophy (Figure 2).

**Figure 3:**
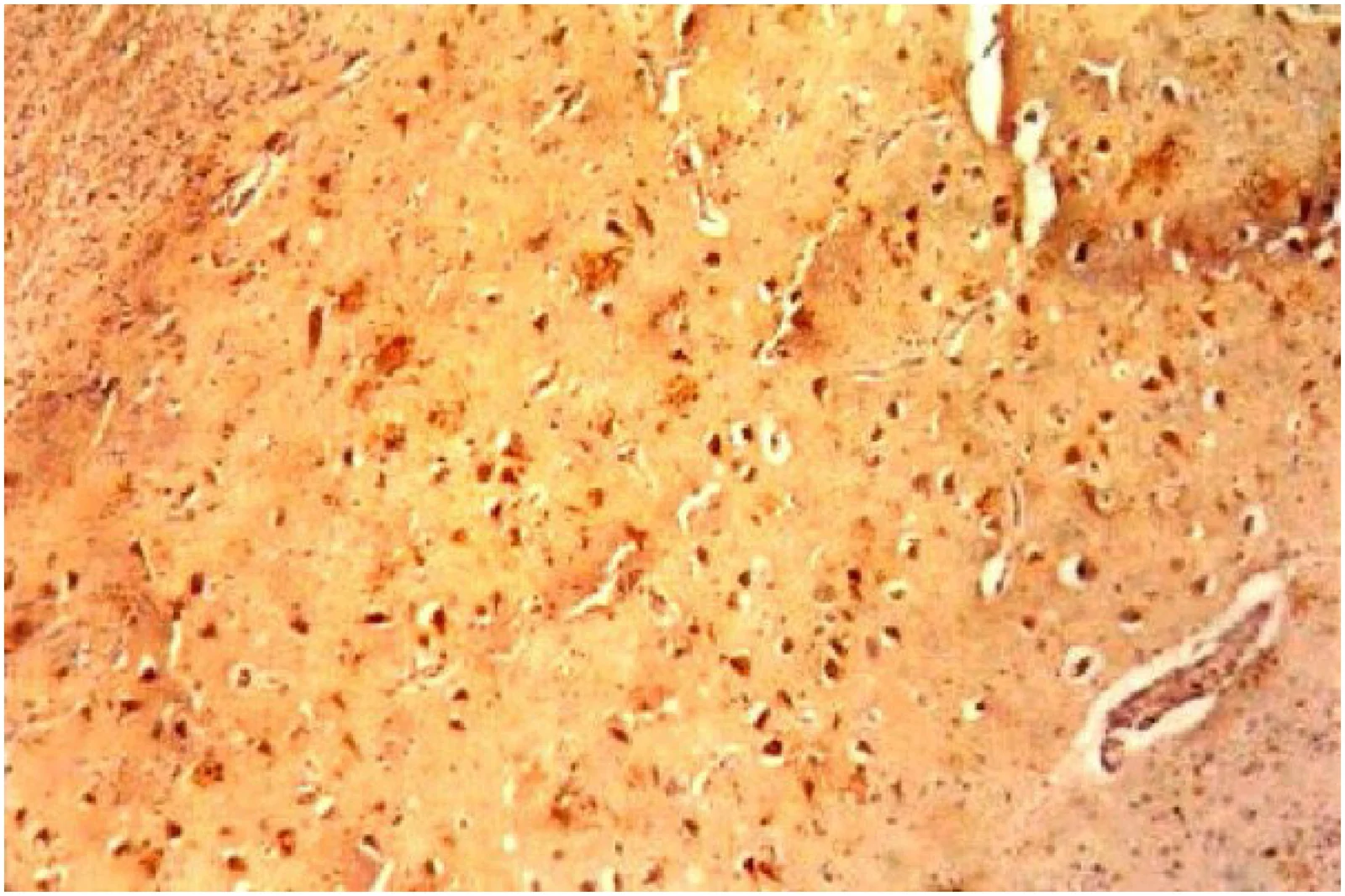
Microscopic study with immunohistochemistry showing the presence of neurofibrillary tangles and senile plaques, each exceeding 20 per 100X.

In a microscopic study, the collected samples included the right and left middle frontal gyri, the right and left superior temporal gyri, the right and left hippocampi, the right and left inferior parietal lobules, the right and left amygdaloid nuclei, and the midbrain. Numerous senile plaques and neurofibrillary tangles were found in all regions^21,22^, except the amygdaloid nuclei, confirmed by immunoreactivity with anti-tau, anti-ubiquitin, and anti-beta-amyloid protein antibodies (Figure 3). There was a marked presence of numerous mature senile plaques, often with perivascular formations. Amyloid deposits were identified in the vascular walls, and lipofuscin deposits were observed in cortical pyramidal neurons. In the hippocampal structures, Hirano bodies were noted, which were also present in the amygdaloid nuclei. No vascular changes or other degenerative changes, such as the presence of cortical Lewy bodies, were observed in the pathological examination. Based on the results of the brain autopsy, the diagnosis of AD was established^20,21^.

## RESULTS

Clinical and functional assessments revealed severe impairment, showing severe deficits on the Pfeffer's scale and the MMSE screening test. Brain CT showed age-appropriate corticosubcortical atrophy, predominantly in the frontal and temporal lobes. SPECT imaging demonstrated moderate-to-severe hypoperfusion in the frontal and limbic regions, including the hippocampus. Postmortem analysis showed abundant senile plaques and neurofibrillary tangles in the relevant brain areas, as assessed by CERAD and Braak criteria, respectively^20,21^.

## DISCUSSION

We report a case of OS in a 54-year-old man who had never previously exhibited such behavior, and who developed delusional jealousy followed by dementia over 27 years, with autopsy confirming a diagnosis of AD. It is possible that the earliest signs of AD were already present two decades before the diagnosis of dementia, through progressive fragmentation of the blood-brain barrier^22^. However, no specific clinical features of AD were yet apparent in the patient's history and instrumental activities of daily life^18,22^, besides delusional jealousy.

It is believed that most cases of Othello Syndrome (OS) occur more frequently in neurological diseases (77%) and in males (62%)^13^. Delusional jealousy has been identified in 15.8%^15^ of patients with AD. Behavioral and psychological symptoms of dementia — particularly hallucinations and delusions — are associated with accelerated cognitive and functional decline, increased risks of hospitalization and mortality, and a greater burden on caregivers^13,14^, but this evolution did not occur in our case. Approximately 50% of patients with dementia exhibit distinct patterns of disease progression and psychosis as clinical manifestations^23^.

The neurobiological factors underlying OS are believed to be heterogeneous and remain poorly explored in the literature, although OS has been reported in a variety of clinical and pathological conditions^6-14^. Nevertheless, the highest prevalence of OS occurs in neurodegenerative diseases (59%)^24^, with Lewy body disease being the most prominent (27%) and AD less frequently represented (5%)^23^. Regardless of the clinical condition associated with OS, the use of dopaminergic agonists in Parkinson's disease, such as ropinirole, pramiprexol, and piribedil, may trigger OS^6-8^. The low prevalence of AD associated with OS strengthens the relevance of the present case, which shows characteristics similar to those described by Alzheimer in 1906^2^.

The pathophysiology of OS is difficult to elucidate for individuals who are not clinically healthy. Delusions of infidelity arise from misinterpretations of everyday events, making it challenging to determine where misinterpretation ends and hallucination begins^25^. Pathological ideas of infidelity occur in neurologically based disorders in at least 30% of cases^26^. Among the neurodegenerative diseases that progress with delirium, the most common are Parkinson's disease^6-8^ and AD^13^. Delirium represents a complex neuropsychiatric manifestation, as affected individuals may develop intense aggression, posing potential risks to the safety of their partners^14,26^.

However, non-neurological causes of OS can also be associated with pregnancy, the postpartum period, or menopause; affected individuals experience insecurity, fear, and the belief that their partner is obtaining sexual gratification elsewhere^27^. Conversely, erectile dysfunction — regardless of its etiology — may give rise to pathological jealousy in men toward their spouses^28^, a behavior not observed in the patient described in this report.

Before being confined to bed for two years until his death, the patient experienced a rapid cognitive decline, while the delusional symptoms of OS gradually diminished as the dementia progressed, and his family increasingly recognized his intellectual impairment. SPECT imaging (Figure 1) revealed moderate hypoperfusion in the frontal region, superimposed on significant frontotemporal cortical atrophy, including the dorsolateral region (Figure 2), similar to findings reported in neuroimaging studies^23^. There was also hypoperfusion in the medial frontal region and in the inferomedial portions of the bilateral temporal lobes, more pronounced on the left. These alterations provide a plausible anatomical-functional basis for the manifestation of delusional jealousy as observed in our case^24^. A neuroimaging study showed greater gray matter loss in the dorsolateral frontal lobe in patients with OS compared with controls^13^.

According to Mendez and Shapira^25^, dysfunction of the medial frontal region may impair inhibitory control and the rational evaluation of affective stimuli, resulting in biased judgments and the formation of affectively charged persecutory beliefs. Fronto-limbic and orbitofrontal circuits, which modulate emotional and social responses, and their hypoperfusion have been associated with loss of insight and a tendency toward emotional confabulation in neurodegenerative conditions. Left mesial temporal impairment, including structures such as the hippocampus and parahippocampal gyrus, may distort the processing of episodic memory and emotional context, leading the patient to reinterpret past experiences in a threatening manner or through the lens of infidelity^24^, as observed with our patient throughout the course of his illness.

This temporolimbic dysfunction, caused by primary atrophy and distortions of the ventricular system, appears to contribute to an abnormal integration of memory, emotion, and relational identity, resulting in delusional jealousy^27^. The association of these findings with the ventricular enlargement observed in the patient's neuroimaging suggests a possible underlying neurodegenerative process, in which the disconnection between frontal cognitive-monitoring regions and temporolimbic structures amplifies affectively driven interpretative distortions. Therefore, it is likely that dysfunction of the frontotemporal circuit, particularly on the left, constitutes the most plausible anatomical substrate for OS.

Among the available treatments, therapy with an antipsychotic combined with an antidepressant is the most recommended (53%)^29,30^. Intranasal oxytocin has also been indicated for the treatment of delusional jealousy^31,32^. However, up to the stage of moderately severe to severe dementia, our patient's delusion was not treated with medication.

## CONCLUSION

A case of OS was reported in a 54-year-old man with Alzheimer's type dementia, representing the first such case published in Brazil. SPECT imaging showed moderate to severe hypoperfusion in the bilateral frontal and limbic regions, accounting for the delusional disorder. The autopsy study revealed cortical atrophy that was most pronounced in the bilateral fronto-temporo-parietal regions. Histopathological examination showed numerous senile plaques and neurofibrillary tangles, confirming AD. It is concluded that the onset of delusional jealousy in an elderly adult man with no previous history of jealousy may be associated with AD.

The main limitation of this study was the lack of more detailed control over how the patient's delusional jealousy progressed before medical follow-up and subsequent neuropsychological monitoring.

## Data Availability

The datasets generated and/or analyzed during the current study are available from the corresponding author upon reasonable request.
